# Cross-National Survey About Nutrition and Nutrition Communication Among Older Adults Aged 60 Years and Above

**DOI:** 10.3390/nu17020315

**Published:** 2025-01-16

**Authors:** Julia Juber, Íris Rafaela Montez De Sousa, Johanna Kreher, Christel Rademacher, Christine Brombach

**Affiliations:** 1 Department Food and Nutrition Sciences, Hochschule Niederrhein University of Applied Sciences, 41065 Moenchengladbach, Germany; christel.rademacher@hs-niederrhein.de; 2 Institute of Food and Beverage Innovation, Zurich University of Applied Sciences, Campus Reidbach, Einsiedlerstrasse 35, 8820 Waedenswil, Switzerlandjohanna_kreher@arcor.de (J.K.); broc@zhaw.ch (C.B.)

**Keywords:** cross-national survey, aging, health, dietary patterns, nutrition communication

## Abstract

**Background/Objectives:** The global population is undergoing a significant demographic shift characterised by an increasing proportion of older individuals. Healthy aging has become a priority for personal well-being and sustainable healthcare systems, with nutrition playing a pivotal role. However, the rise in non-communicable diseases (NCDs), malnutrition, and a shift in eating behaviour underscore the need for tailored, effective nutrition communication strategies. This research is intended to provide the basic data needed to support the development of tailored nutritional communication strategies and practices. **Methods:** To investigate these aspects, a cross-national survey about nutrition and nutrition communication was conducted within the framework of the Innovative Training Network SmartAge, focusing on older adults aged 60 years and above in Germany, Switzerland, Spain, and France (each 25%; N = 1000 persons). This paper specifically focuses on the development, methodology, and discussion of the survey and aims to investigate the characteristics of the sample in relation to their dietary patterns and food choices within the European context. **Results:** The analysis identified significant associations between various plant- and animal-based food items and the variables gender, age group, and country. Spanish participants showed a dietary pattern towards the Mediterranean Diet, while German participants showed tendencies towards the Western Diet. Furthermore, the findings revealed that protein-rich foods such as meat, fish, nuts and seeds, legumes, and (whole) grains were infrequently consumed, particularly among German and Swiss participants. **Conclusions:** This study highlights the need to promote adequate protein intake among older adults, emphasising diverse sources like legumes, nuts and seeds, (whole) grains, and lean meats to support healthy aging. For the development of future nutrition communication strategies, the influence of the specific social, cultural, and traditional factors needs to be considered.

## 1. Introduction

The global population is currently experiencing a significant demographic megatrend known as population aging [1]. This phenomenon is characterised, among many other factors, by the increasing number of people reaching an advanced age, leading to a steady growth in the proportion of older individuals [2]. The share of adults aged 65 years and above is currently the highest in Europe and the United States, at around 19%, and is projected to grow to 26.9% by 2050 [2]. With increased age, individuals face a higher risk of functional limitations, illness, and reduced mental and physical capacity, necessitating additional healthcare support [3,4]. Consequently, healthcare expenditures have risen significantly, especially in the age group of very old individuals from 80 years onwards [4,5,6,7]. Moreover, population aging contributes to a diminishing labour force and an imbalanced dependency ratio, as there are fewer individuals of working age compared to the population of older adults [8].

The persistent and escalating rise of non-communicable diseases (NCDs) is characterised by chronic conditions and accounts for 74% of the global deaths [9]. Unhealthy lifestyle factors, such as an inadequate diet, physical inactivity, tobacco use, excessive alcohol consumption, and air pollution, contribute to NCDs across all age groups. Overweight and obesity are also contributing to this rise [9,10]. The risk and concern for malnutrition is also increased, particularly in the elderly [11,12,13,14].

Considering this, healthy aging has emerged as a goal for individuals and policymakers, by promoting personal well-being, independence, and a stable healthcare system. Nutrition plays a crucial role, as numerous studies have highlighted its positive impact on aging, morbidity, and mortality [15,16,17,18,19,20]. Moreover, eating behaviour and food choices also change during the aging process [21]. Hence, tailored and effective nutrition interventions and communication strategies based on the eating behaviours and food choices of the elderly are required, specifically considering the target groups’ historical and cultural conditioning over the lifespan. Nutrition and healthcare communication play pivotal roles in raising awareness, providing education, and encouraging healthier food choices. To achieve this, messages have to be scientifically sound and easily understandable for the intended audience [22,23]. Additionally, given the heterogeneity of the older adult population and the various health issues they may face, nutrition and health-related communication needs to be multifaceted to cater to individual needs, as “one size does not fit all” [24] (p. 113).

This study was conducted with funding provided by the Marie Skłodowska-Curie Innovative Training Network (ITN) SmartAge [25]. The primary objective of SmartAge is to advance multidimensional knowledge related to older adults. To specifically explore the domain of nutrition and nutrition communication of the elderly, the authors developed a comprehensive, cross-national survey, which was distributed to older adults aged 60 years and above across Germany, France, Switzerland, and Spain. While the broader aim of this sub-project is to design evidence-based, tailored strategies and practices, this paper focuses on the development and methodology of the questionnaire as well as the demographic characterisation of the sample. Furthermore, this study conducts an exploratory, descriptive analysis of nutritional behaviours and food choices within the European context, using Pearson’s Chi-square test to examine associations between the variables gender, age group, and country. This study does not provide concrete actions or practices.

Based on the literature, the authors hypothesise that (1) gender, (2) age group, and (3) country influence specific nutritional preferences, food choices, and behaviours driven by social, cultural, and dietary traditions as well as national and geographical factors. Knowing these in detail is a prerequisite for developing nutritional communication for this target group.

## 2. Materials and Methods

### 2.1. Study Design and Participants

The study design employed in this research was a standardised survey using a web-based online questionnaire. The survey was distributed in Germany, Switzerland, Spain, and France through an online panel provided by a market research agency, with distribution funding provided by ITN SmartAge. Participants were required to be at least 60 years old, be proficient in one of the respective national languages, live independently in a non-institutionalised setting in one of the selected countries, and have internet access and an appropriate device. No restrictions were placed on income, ethnicity, or religion. Individuals who did not fulfil any of the previous requirements were excluded from the survey.

The minimum sample size was determined using the calculation method described by Daniel and Cross [26], considering a confidence interval of 95%, a margin of 5%, and a *p*-value of 0.05, which resulted in a minimum requirement of 385 participants. However, the sample size was increased to 1000 participants to enhance the statistical power by reducing the risk of Type II errors and providing more accurate effect estimates [27]. Additionally, the larger sample size supports robust exploratory subgroup analyses, allowing for meaningful comparisons across different demographic groups while maintaining sufficient statistical power [28]. A target quota of 250 participants per country was set.

### 2.2. Ethical Considerations

Ethical considerations were upheld throughout the research process. All participants were informed about the purpose of the study, the voluntary nature of participation, and the confidentiality of their data. Informed consent was obtained from all participants at the beginning of the survey. Personal information was treated with strict confidentiality, and the data were analysed and reported in aggregated form to maintain anonymity. Furthermore, ethical considerations were followed during the questionnaire development, guided by the Federal Act on Research involving Human Beings [29] and the ICC/ESOMAR International Code on Market, Opinion, and Social Research and Data Analytics [30]. A declaration of consent was obtained from participants to elicit, process, and use their personal data. An ethical compliance submission was made to the Swiss Ethics Committee, although it was determined that the research project did not fall within the scope of the Human Research Act, and authorisation was not required.

### 2.3. Methods

The questionnaire was developed in English and translated into the national languages of the countries included (German, French, Spanish, and Italian) by native speakers. The questions were formulated precisely, and answer designs were standardised as they consisted primarily of single or multiple-choice scales. To ensure the questionnaire was suitable for the target group and to minimise participant burden, open-ended questions were limited, as writing on a computer or smartphone keyboard may be challenging for older participants.

In total, the questionnaire encompassed a set of 30 inquiries and included several sections such as socio-demographic information, dietary patterns, health status, information sources, and information-seeking behaviour as well as motives, wishes, and needs related to nutrition and health. For the scope of this publication, the socio-demographic information and the evaluation of dietary patterns have been subjected to statistical analysis.

#### 2.3.1. Socio-Demographic Information and Lifestyle

The demographic information included age, gender, and highest level of education (adapted to country). One question was asked about regular activities (Reading; Playing games; Crossword or Sudoku; Painting or Drawing; Meeting with friends, family, neighbours; Gardening; Leisure or Club sports; Walking or Hiking; Riding a bike; None of these). In addition, there were five questions about the living conditions such as country (Germany; Spain; France; Switzerland), environmental description (Rural; Urban or suburban), total number of persons living in the household (1; 2; 3; 4; 5; 6 or more), responsibility for cooking and grocery shopping (Each: Yes; No; Shared responsibility; External support), and places for grocery shopping (Specific grocery store; Retail trade; Healthy/organic food store; Weekly market or producer; None of these).

#### 2.3.2. Dietary Patterns

Dietary patterns were evaluated using consumption frequencies of plant-based (Vegetables; Legumes; Fruits and berries; Nuts and seeds; Vegetable oils; (Whole) grains and products; Alcohol) and animal-based (Dairy and dairy products; Butter/margarine; Fish (not fried); Poultry (not fried); Red meat and products; Pastries and sweets; Fast food or fried food) food items. The respondents could rank their frequencies on a four-point Likert scale from Daily (=1); More than once a week (=2); Less than once a week (=3); to Rarely or never (=4).

### 2.4. Pre-Test

A comprehensive pre-test of the questionnaire was conducted to optimise its wording and the answer design and identify any ambiguities or comprehension issues. The pre-test involved independent individuals from the target group of the respective countries who completed the questionnaire online under everyday survey conditions. The questionnaire underwent two phases of feedback, resulting in minor changes based on further feedback.

### 2.5. Data Collection

The data collection was conducted by the market research agency “IMR Institute for Marketing Research GmbH, Frankfurt, Germany”. Quota sampling was employed to ensure a representative sample. The quotas were set based on demographic variables such as age, gender, and country to match the population distribution of each country. Gender quotas were set at 50% female and 50% male. Age quotas were set at 60–70 years (60%), 70–80 years (30%), and 80+ years (10%). For all countries, the quotas were set at 250 persons. For further information, see Supplementary Materials Table S1.

The online panel provided by the market research agency was used to recruit participants who met the inclusion criteria.

The data collection took place between 29 November 2022 and 7 December 2022. The survey was a non-recurring process and was hosted on a secure online platform, which ensured the confidentiality and anonymity of the participants’ responses. The aggregate count of questionnaire distributions reached 2187 recipients. Among these, 114 individuals (5%) were excluded due to non-compliance with inclusion criteria, 193 individuals (9%) discontinued participation and were excluded for incomplete or unusable records, and 880 individuals (40%) were declined admission as the pre-established target quotas were met. Consequently, 1187 people did not meet the inclusion criteria or were excluded from participation, resulting in an effective response rate of 46% for this quota-based sample.

### 2.6. Statistical Analysis

The collected data underwent thorough processing by the market research agency, involving acquisition, review, and transformation. A data screening eliminated incomplete or unusable records. The statistical software IBM SPSS Statistics 27 and Excel were employed for data analysis. Descriptive statistics and inferential statistics, including the Pearson’s Chi-square test, were conducted, with interpretation based on the Fisher’s exact test *p*-value if the assumptions of the Pearson’s Chi-square test were not fulfilled [31,32]. The chosen significance level was set at 5% (α = 0.05), warranting rejection of the null hypothesis for outcomes where *p* < α. The hypotheses were set as follows:

Null Hypothesis (H_0_):

There is no association between the categorical variables. The distribution of one variable is independent of the other variable(s).

Alternative Hypothesis (H_1_):

There is an association between the categorical variables. The distribution of one variable depends on the other variable(s).

At the beginning of statistical analysis, two new variables were generated by categorising age into three age groups (60–64 years, 65–74 years, or 75 years and older), and educational attainment levels into four categories (no school education, primary, secondary, or tertiary education).

## 3. Results

### 3.1. Evaluation of Socio-Demographic Information and Lifestyle

The dataset of 1000 participants was analysed, with an equal representation across gender and countries. The distribution of the socio-demographic information is presented in Table 1. The mean age of the participants was 68 years, with a range from 60 to 91 years, yielding a 31-year age span. The median age was 67 years, and the standard deviation was 5.82. Therefore, about half of the participants fell within the age range of 65 to 74 years.

Regarding educational attainment, the majority of participants reached the secondary level, encompassing primary school, general school education, and initial vocational training. Furthermore, almost one-third held tertiary qualifications, comprising Bachelor’s, Master’s, or Doctorate degrees. A small percentage had no school education or only completed primary school.

The top three regularly undertaken activities by participants were walking or hiking, reading, and socialising with, e.g., friends, family, or neighbours. Consequently, the respondents consistently participated in a mix of physical, mental, and social activities. Further activities are listed in Table 1.

The evaluation of living conditions showed that more than half of the participants shared their living space with one other person. Nearly two-thirds characterised their surroundings as either urban or suburban, with the remaining one-third reporting rural living conditions.

Regarding the examination of culinary and procurement activities, approximately 60% of the study participants were responsible for both cooking and conducting grocery shopping. External support was requested only to a minor extent. With respect to grocery shopping habits, almost every participant routinely visited supermarkets. Additionally, almost half of the participants acquired groceries from specific stores such as butchers or bakeries. Organic food stores were frequented by the smallest proportion of participants.

### 3.2. Evaluation of Dietary Patterns

The analysis of dietary patterns of survey participants was conducted in four steps. Initially, the average consumption frequencies of plant-based and animal-based food items were analysed using the median and mean to assess the central tendency of the sample. Subsequently, the evaluation of these dietary patterns and their association with gender, age groups, and across the countries was performed. The *p*-values of the Pearson’s Chi-square test and Fisher’s exact test of this analysis are presented in Table 2.

#### 3.2.1. Consumption Frequencies of the Sample

Plant-based food items

**Figure 1:**
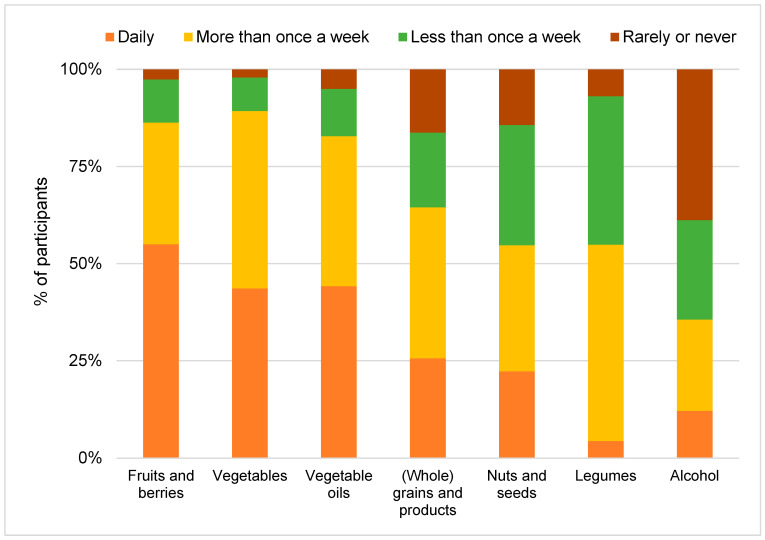
Consumption frequencies of plant-based food items (1 = daily, 2 = more than once a week, 3 = less than once a week, 4 = rarely or never).

Fruits and berries emerged as the most frequently consumed food group, with a median consumption frequency of 1, indicating that more than half of the participants consumed these items on a daily basis. Conversely, the other five food items, including vegetables, vegetable oils, (whole) grains and products, nuts and seeds and legumes, exhibited a median consumption frequency of 2, suggesting a central tendency of consumption more than once a week. Notably, alcohol consumption occurred at a markedly lower frequency, with a median of 3, indicating consumption less than once a week. Figure 1 illustrates the distribution of plant-based food items.

Animal-based food items

**Figure 2:**
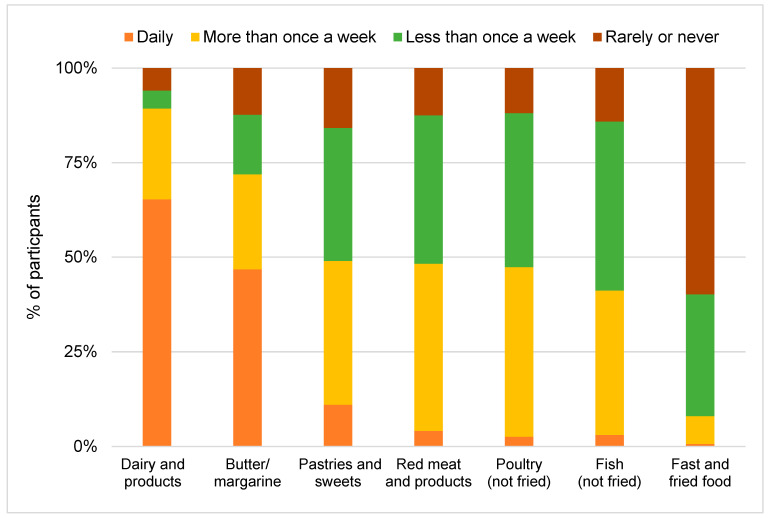
Consumption frequencies of animal-based food items (1 = daily, 2 = more than once a week, 3 = less than once a week, 4 = rarely or never).

The results of the consumption frequencies of animal-based food items are illustrated in Figure 2. Specifically, dairy and dairy products solely exhibit a median consumption frequency of 1, signifying prevalent daily consumption among participants. Additionally, butter or margarine demonstrated a median value of 2, indicating a consumption occurring more than once a week. Conversely, pastries and sweets, red me at and products, poultry, and fish, along with fast food or fried food, resulted in higher median values of 3 and 4, implying less frequent consumption.

#### 3.2.2. Association Between Dietary Patterns and Gender

Plant-based food items

Significant associations between gender and the consumption frequencies of plant-based food items were detected in this study. Noteworthy disparities were found for vegetables (Pearson Chi^2^(3) = 26.270; *p* < 0.001), fruits and berries (Pearson Chi^2^(3) = 14.897; *p* = 0.002), vegetable oils (Pearson Chi^2^(3) = 11.409; *p* = 0.010), legumes (Pearson Chi^2^(3) = 12.146; *p* = 0.007), and alcohol (Pearson Chi^2^(3) = 40.466; *p* < 0.001). The analysis showed that women exhibited higher daily consumption rates of vegetables and fruits and berries compared to men. Moreover, women predominantly consumed vegetable oils daily, whereas most men reported using them several times a week. Conversely, men demonstrated more frequent consumption of legumes and alcohol.

Animal-based food items

In terms of animal-based food items, significant associations were revealed for red meat and products (Pearson Chi^2^(3) = 24.019; *p* < 0.001) and fast food or fried food (Fisher’s exact test = 9.212; *p* = 0.025). Men reported a more frequent consumption of red meat and products compared to women, while women displayed a greater tendency towards rare or negligible consumption of these items relative to men. A similar trend was observed in the consumption frequencies of fast or fried food, with men displaying slightly higher consumption rates compared to women. Based on these results, we reject the null hypothesis (H_0_) and accept the alternative hypothesis (H_1_), indicating formal significant associations between gender and dietary patterns.

#### 3.2.3. Association Between Dietary Patterns and Age Groups

Plant-based food items

The test indicated significant associations between the consumption of vegetables (Pearson Chi^2^(6) = 18.561; *p* = 0.005) and fruits and berries (Pearson Chi^2^(3) = 15.383; *p* = 0.017) with age groups. Notably, the oldest age group of participants aged 75 years and above reported higher daily consumption rates of both vegetables and fruits and berries compared to the younger age groups. Based on these results, we reject the null hypothesis (H_0_) and accept the alternative hypothesis (H_1_), indicating formal significant associations between age groups and the consumption of these plant-based food items.

Animal-based food items

In contrast, no significant association was found between the consumption of animal-based food items and age groups. Thus, we retain the null hypothesis and assume no formal statistical associations.

#### 3.2.4. Association Between Dietary Patterns and Countries

Plant-based food items

**Figure 3:**
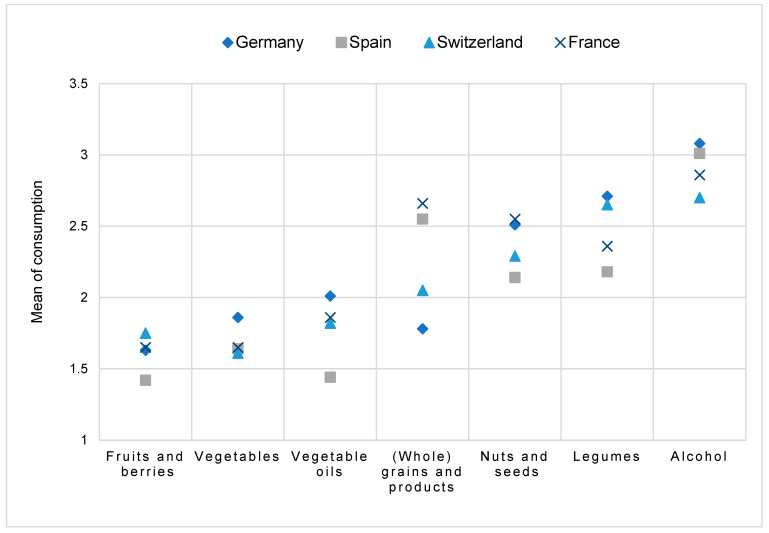
Mean consumption frequencies of plant-based food items across the countries (1 = daily, 2 = more than once a week, 3 = less than once a week, 4 = rarely or never).

The examination of cross-national differences in food consumption was a focal point of this study. By applying the Pearson’s Chi-square test, the analysis revealed significant differences (*p* < 0.001) in the consumption patterns of all seven plant-based food items across the countries. To present the comprehensive results of this cross-national comparison, the mean consumption frequency was employed, acknowledging that this averaging approach is less suitable for ordinal variables. However, given the limited value range of consumption frequency (1 = daily, 2 = more than once a week, 3 = less than once a week, 4 = rarely or never), the use of the median seemed less appropriate as well. Despite its limitations, the mean provides an impression of the countries’ consumption patterns. Figure 3 depicts the mean consumption of plant-based foods across the countries. Notably, values close to 1 indicate daily intake, whereas values near 4 signify rare or negligible consumption. Consequently, as the mean increases, the frequency of consumption decreases.

Figure 3 shows that fruits and berries as well as vegetables emerged as the most commonly consumed items across all countries. Spain exhibited particularly high consumption rates for fruits and berries, with a substantial portion of participants reporting daily intake. Conversely, vegetable consumption appeared to be slightly lower in Germany compared to the other surveyed countries. Interestingly, Spanish participants displayed remarkably higher consumption frequencies for vegetable oils compared to the other countries. The same trend occurred for legumes and nuts and seeds. Similarly, it is noteworthy that German participants reported the highest consumption frequency for (whole) grains and products, indicating a distinct dietary preference compared to Spain and France, where consumption frequencies for this food group were comparatively lower. Alcohol intake was the least frequent among all plant-based food items analysed. Switzerland showed the most frequent consumption in comparison to the other three countries, but the consumption was less than once a week on average across all countries.

Animal-based food items

**Figure 4:**
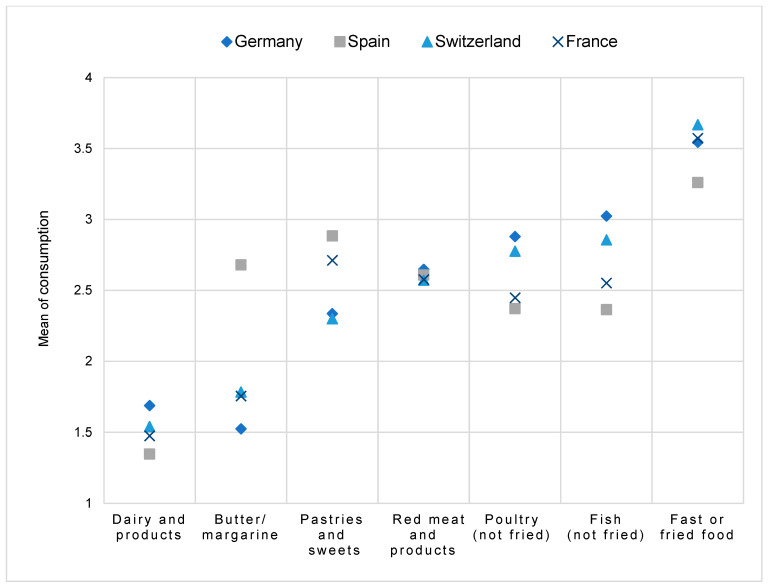
Mean consumption frequencies of animal-based food items across the countries (1 = daily, 2 = more than once a week, 3 = less than once a week, 4 = rarely or never).

Regarding animal-based food items, the Pearson’s Chi-square test could identify significant associations for all food items (*p* < 0.001), except for the consumption of red meat and products. Figure 4 depicts the mean consumption of animal-based food items across the countries.

Spanish participants showed higher consumption frequencies for dairy and dairy products compared to German participants. Conversely, German participants consumed butter or margarine more frequently than those in Spain, where consumption was significantly reduced. Consumption patterns for pastries and sweets were similar between Germany and Switzerland, while France and Spain displayed lower consumption frequencies. Red meat and products showed no significant differences across countries. Poultry and fish were more frequently consumed by Spanish and French participants compared to German and Swiss participants. Particularly noteworthy was the high proportion of daily fish consumption in Spain. Finally, fast food or fried food was consumed relatively rarely across all countries, with slightly higher intake observed among Spanish participants compared to the others. Based on these results, we reject the null hypothesis (H_0_) and accept the alternative hypothesis (H_1_), indicating formal significant associations between countries and dietary patterns.

The comparison of consumption frequencies between plant-based and animal-based food groups revealed that plant-based items were consumed more frequently, with a median value predominantly at 2. Conversely, most animal-based food groups had a median of 3, except for fast food or fried food, which had a median of 4. Additionally, similar dietary patterns were observed between Germany and Switzerland as well as between Spain and France, especially in terms of the mean consumption of animal-based foods. The analysis indicated a statistical association between dietary patterns and gender, age groups, and countries, which underscores the complex interplay of social, cultural, and regional factors in shaping dietary preferences and consumption patterns across European nations.

## 4. Discussion

The results of this survey regarding the socio-demographic questions showed a high level of education among the participants, indicating that a significant proportion of them were financially secure, as higher education levels are associated with higher income [33,34]. Furthermore, most participants were physically and mentally active, regularly engaged in social interactions, and were primarily responsible for cooking and grocery shopping. These factors have a positive association with healthy aging [10]. The low reliance on external food suppliers further demonstrates their independence and self-determination in lifestyle choices. Furthermore, it is worth noting that the majority of participants lived in (sub)urban areas, which may have provided them with easy access to daily essentials.

The dietary patterns of the sample were assessed primarily through consumption frequencies, which limited the ability to make comprehensive qualitative and quantitative evaluations and comparisons of nutritional intake. Therefore, final conclusions about the adequacy of participants’ diets could not be drawn. Although the sample was extensive, the heterogeneous nature of the elderly population requires careful consideration when generalising the findings. However, according to respective national consumption guidelines [35,36,37,38], a substantial proportion of participants did not meet the recommended intake for several food groups, especially fruits, vegetables, and legumes. On the other hand, a significant majority of participants consumed dairy and dairy products in accordance with the guidelines. Notably, the consumption of protein-rich sources such as red meat, fish, and poultry was relatively low. This may indicate potential dietary insufficiencies in protein intake. Although most participants included dairy and dairy products in their daily diet, their consumption of (whole) grains and products, nuts and seeds, and legumes was limited. This underscores the need to promote a diverse protein intake. The low consumption of protein in Germany and Switzerland is particularly noteworthy, despite the fact that studies by Rempe et al. and Bauer et al. highlighted the importance of and deficits in protein intake in older individuals [39,40].

The analysis revealed gender-dependent consumption patterns for both plant-based and animal-based food items. Women showed a more frequent consumption of vegetables, fruits and berries, and vegetable oils than men, while men consumed legumes and alcohol more often. Men were also more likely to consume red meat and products and fast food or fried food. These findings are also reflected in the data collected by Forsa for the German Nutrition Report 2022 and 2023, which show higher intakes of fruits and vegetables among women [41,42]. Furthermore, gender disparities in food consumption, particularly in fruit, vegetable, and meat intake, have been substantiated by a cross-sectional study across 21 European countries conducted by Stea et al. and research from the Survey of Health, Ageing, and Retirement (SHARE) in Europe [43,44]. Similarly, the global status report on alcohol and health by the World Health Organization (WHO) found that men are more likely to drink alcohol more often and in greater quantities than women [45]. These differences in gender suggest that women have a greater awareness of nutrition and health, as their dietary patterns are more aligned with the recommendations of national nutrition societies for a balanced diet, rich in fruits, vegetables, and lean proteins [46].

Nutritional and health awareness may also be a driver for age-related differences in food choices. Studies have shown that increased consumption of fruits and vegetables is associated with longevity and reduced mortality, indicating a health-conscious motive among older individuals [47,48,49]. These studies support the idea that health awareness is a significant motivator for healthy eating among the elderly [39,50,51]. This may explain the more frequent daily consumption of vegetables and fruits and berries among the oldest participants compared to the younger age groups. The analysis of dietary patterns across countries revealed significant results for all food items except red meat and its products. Spain’s dietary profile was characterised by a frequent intake of fruit and berries, vegetable oils, nuts and seeds, legumes, poultry, and fish consumption, indicating an adherence to the Mediterranean Diet (MedDiet) prevalent in the region. This is in line with existing literature that emphasises the nutritional advantages and health benefits of the MedDiet, including a decreased risk of cardiovascular diseases and improved longevity [52]. In contrast, Germany’s dietary pattern was more consistent with the Western diet, which is characterised by a lower consumption of vegetables, unrefined vegetable oils, nuts and seeds, and legumes but higher intakes of grains and grain products [53]. The difference between the observed MedDiet pattern and the Western pattern in Germany may be due to cultural preferences, especially in bread consumption. Germany has a rich bakery culture, with a wide variety of breads that incorporate whole grains, rye, or spelt, in contrast to the more refined wheat flour-based baked items commonly consumed in France and Spain [54,55]. Furthermore, the dietary habits of respondents from Germany and Switzerland indicate a serious lack of meat and fish consumption. This emphasises the need to diversify protein sources among this demographic group. These findings support the study by Rempe et al., which highlights inadequate protein intake among older German adults. Therefore, promoting alternative protein sources such as legumes and nuts is imperative to ensure a sufficient diet [39]. Additionally, Switzerland had a slightly higher rate of alcohol consumption. This could be due to sociocultural factors or differences in lifestyle habits. In contrast, Spain had a higher frequency of fast food or fried food consumption, which is likely influenced by urban demographics and the greater availability of such food options in urban areas compared to rural regions. These findings highlight the importance and influence of health awareness and motivation as well as cultural, geographic, and socioeconomic factors in shaping food choices and nutritional habits. Therefore, this paper emphasises the necessity of tailored and targeted nutrition communication strategies to promote healthier and age-related dietary patterns and prevent malnutrition in the elderly.

Lastly, it needs to be remarked that, while this study offers a valuable and up-to-date overview of dietary patterns among the elderly in parts of Europe, providing critical insights into their food choices, several limitations should be acknowledged. When interpreting a survey, it should be noted that the results are not taken as conclusive evidence but rather as an indication of potential formal statistical significance. In this context, it is important to clarify that Pearson’s Chi-square test is a measure of general association and a test of independence. Given the repeated application of the Pearson’s Chi-square test, the potential inflation of the Type I error rate should be considered when interpreting the results [32]. To address this limitation, future analyses should apply corrections such as the False Discovery Rate or the more conservative Bonferroni adjustment to ensure robust interpretations.

Furthermore, conducting a standardised online questionnaire proved to be an appropriate study design, as it allowed for a thorough understanding of the target group of adults aged 60 years and above across four European countries. However, the online survey methodology introduced biases, including selective participant recruitment and coverage error, as it relied on internet access, potentially excluding older adults who are not familiar with digital technology [56]. Although this exclusion could be mitigated by the increasing technology adoption among the elderly [57], the relatively low representation of participants aged 75 years and older and the use of mean values for ordinal data instead of medians also limited the analysis. Additionally, it is important to acknowledge that women represent a larger share of the national populations within this age group. Although this analysis did not employ post-stratification adjustments with survey weights, future studies should consider this factor to enable more gender-balanced comparisons. Nevertheless, this study offers current and advanced research insights, as comparing the findings with national assessments and other studies proved challenging due to outdated datasets, variations in methodologies, and inconsistencies in food group or age group categorisations, which hindered cross-study comparability.

## 5. Conclusions

This study comprehensively explored the characteristics and dietary patterns of older adults aged 60 years and above from Germany, Switzerland, Spain, and France, emphasising the importance of focusing on this demographic group. The survey methodology, employing a standardised quantitative approach via an online questionnaire, proved suitable for gathering extensive data across several European countries. The robustness of this study was strengthened by the large sample size and successful quota sampling. This study was able to contribute to a clearer understanding of the dietary patterns of older adults in European countries while offering insights into areas requiring improvement to design evidence-based nutrition communication strategies. These insights are of particular relevance to stakeholders, policymakers, and experts in the field for deriving concrete nutrition recommendations within the target group.

In more detail, the analysis revealed gender-, age-, and country-specific differences in dietary patterns, reflecting social, cultural, and traditional influences. It underscored the critical importance of ensuring and promoting adequate protein intake among older adults, particularly through sources such as legumes, nuts and seeds, (whole) grains, and lean meats. Promoting the consumption of plant-based foods in general could be identified as a key strategy for improving dietary quality in this age group. For clinical practice, the findings emphasise the value of personalised dietary recommendations, tailored by gender and age. Public health initiatives should prioritise education on protein-rich and plant-based diets, such as legumes, nuts, seeds, and whole grains, to support healthier eating habits and prevent malnutrition in older age. Moreover, it will be crucial to adapt nutrition communication strategies to the food culture and conditions of each country for effective nutritional education.

Future research endeavours should extend these findings by investigating nutrition in both quantitative and qualitative dimensions to close knowledge gaps and deepen the understanding on living conditions in respective everyday life contexts. This study provides the foundation for further research on the diverse topics addressed in the questionnaire, which advances the development of effective nutritional communication strategies and practices tailored to the needs of older adults at both national and European levels.

## Figures and Tables

**Table 1:** Socio-demographic and general information of the sample (N = 1000).

Variable	Number of Participants	% of Participants
**Gender**		
Female	500	50%
Male	500	50%
**Country**		
Germany	250	25%
France	250	25%
Switzerland	250	25%
Spain	250	25%
**Age group**		
60–64 years	329	32.9%
65–74 years	518	51.8%
75 years and above	153	15.3%
**Educational level**		
No school education	11	1.1%
Primary level	45	4.5%
Secondary level	636	63.6%
Tertiary level	308	30.8%
**Regular activities**		
Walking or hiking	710	71.0%
Reading	675	67.5%
Socialising with, e.g., friends, family, or neighbours	639	63.9%
Crossword puzzles or sudokus	447	44.7%
Gardening	401	40.1%
Playing games	353	35.3%
Riding a bike	239	23.9%
Leisure sports or club sports	198	19.8%
Painting or drawing	83	8.3%
**Persons per household**		
1 person	276	27.6%
2 persons	543	54.3%
3 persons	109	10.9%
4 persons	52	5.2%
5 persons	15	1.5%
6 or more persons	5	0.5%
**Environmental description**		
(Sub-)urban	639	63.9%
Rural	361	36.1%
**Places for grocery shopping**		
Supermarket	945	94.5%
Specific stores (e.g., butcher)	458	45.8%
Weekly market or producer	244	24.4%
Organic food stores	89	8.9%

**Table 2:** *p*-Values of Pearson’s Chi-square test and Fisher’s exact test analysis between dietary patterns and gender, age groups, and countries. Significance level = 5% (α = 0.05).

Food items	Gender	Age Group	Country
Fruits and berries	0.002	0.017	<0.001
Vegetables	<0.001	0.005	0.001
Vegetable oils	0.010	0.824	<0.001
(Whole) grains and products	0.067	0.827	<0.001
Nuts and seeds	0.064	0.460	<0.001
Legumes	0.007	0.478	<0.001
Alcohol	<0.001	0.063	0.001
Dairy and products	0.547	0.664	<0.001
Butter/margarine	0.892	0.214	<0.001
Pastries and sweets	0.057	0.887	<0.001
Red meat and products	<0.001	0.126	0.073
Poultry (not fried)	0.066	0.603	<0.001
Fish (not fried)	0.068	0.098	<0.001
Fast food or fried food	0.025 *	0.336	<0.001

* Fisher’s exact test.

## Data Availability

Data are unavailable due to privacy or ethical restrictions.

## Supplementary Materials

The following supporting information can be downloaded at: https://www.mdpi.com/article/10.3390/nu17020315/s1, Table S1: Quotas and inclusion and exclusion criteria for data collection; Table S2: Results of consumption frequencies of plant- and animal-based food items; Table S3: Association between dietary patterns and gender; Table S4: Association between dietary patterns and age groups; Table S5: Association between dietary patterns and countries; Table S6: Mean of consumption across the countries; Figure S1: Questionnaire (General English version).
