# Supplementary material for: Cross-National Survey About Nutrition and Nutrition Communication Among Older Adults Aged 60 Years and Above
Source: Nutrients. 2025 Jan 16;17(2):315. doi: 10.3390/nu17020315 (PMC11768146; doi:10.3390/nu17020315)
Supplement: Supplementary file 1 [file nutrients-17-00315-s001.zip › nutrients-3398191-supplementary.pdf]

Supplementary materials

Table S1: Quotas, inclusion and exclusion criteria for data collection.

Quotas	N (% of N)
Total participants	1,000 (100%)
**Countries**	
Germany	250 (25%)
France	250 (25%)
Spain	250 (25%)
Switzerland	250 (25%)
**Gender**	
Female	500 (50%)
Male	500 (50%)
**Age**	
60 - 69 years	600 (60%)
70 - 80 years	300 (30%)
80+ years	100 (10%)

Inclusion criteria

The participant needs to be:

- consenting the participation in the survey
- at least 60 years old
- proficient in one of respective national languages (German, French, Italian, Spanish)
- living independently in a non-institutionalised setting in one of the selected countries
- connected to the Internet and be able to use an appropriate device
- completing the survey

Exclusion criteria

The participant:

- does not agree to take part in the survey
- is younger than 60 years
- is not proficient in one of respective national languages (German, French, Italian, Spanish)
- does not live independently in a non-institutionalised setting in one of the selected countries
- is not connected to the Internet and is not able to use an appropriate device
- does not complete the survey / stopped participation = incomplete data set
- takes part in survey and the corresponding quotas are already complete

Table S2: Results of consumption frequencies of plant- and animal-based food items.

	Daily	More than once a week	Less than once a week	Rarely or never	Median	Mean
**Food items**						
Fruits and berries	55.0%	31.3%	11.1%	2.6%	1	1.61
Vegetables	43.6%	45.7%	8.6%	2.1%	2	1.69
Vegetable oils	44.2%	38.6%	12.2%	5.0%	2	1.78
(Whole) grains and products	25.6%	38.9%	19.2%	16.3%	2	2.26
Nuts and seeds	22.3%	32.4%	31.0%	14.3%	2	2.37
Legumes	4.4%	50.5%	38.2%	6.9%	2	2.48
Alcohol	12.1%	23.5%	25.6%	38.8%	3	2.91
Dairy and products	65.3%	24.0%	4.8%	5.9%	1	1.51
Butter / margarine	46.8%	25.1%	15.8%	12.3%	2	1.94
Pastries and sweets	11.0%	38.0%	35.2%	15.8%	3	2.56
Red meat and products	4.1%	44.2%	39.2%	12.5%	3	2.60
Poultry (not fried)	2.6%	44.8%	40.7%	11.9%	3	2.62
Fish (not fried)	3.0%	38.2%	44.7%	14.1%	3	2.70
Fast food or fried food	0.7%	7.3%	32.2%	59.8%	4	3.51

Table S3: Association between dietary patterns and gender.

	Female<br>*n*=500 (%)	Male<br>*n*=500 (%)	Total<br>*N*=1,000 (%)
**Plant-based food items**			
Fruits and berries			
Daily	303 (60.6%)	247 (49.4%)	550 (55.0%)
More than once a week	133 (26.6%)	180 (36.0%)	313 (31.3%)
Less than once a week	49 (9.8%)	62 (12.4%)	111 (11.1%)
Rarely or never	15 (3.0%)	11 (2.2%)	26 (2.6%)
Vegetables			
Daily	257 (51.4%)	179 (35.8%)	436 (43.6%)
More than once a week	199 (39.8%)	258 (51.6%)	457 (45.7%)
Less than once a week	33 (6.6%)	53 (10.6%)	86 (8.6%)
Rarely or never	11 (2.2%)	10 (2.0%)	21 (2.1%)
Vegetable oils			
Daily	241 (48.2%)	201 (40.2%)	442 (44.2%)
More than once a week	168 (33.6%)	218 (43.6%)	386 (38.6%)
Less than once a week	62 (12.4%)	60 (12.0%)	122 (12.2%)
Rarely or never	29 (5.8%)	21 (4.2%)	50 (5.0%)
(Whole) grains and products			
Daily	146 (29.2%)	110 (22.0%)	256 (25.6%)
More than once a week	189 (37.8%)	200 (40.0%)	389 (38.9%)
Less than once a week	90 (18.0%)	102 (20.4%)	192 (19.2%)
Rarely or never	75 (15.0%)	88 (17.6%)	163 (16.3%)
Nuts and seeds			
Daily	127 (25.4%)	96 (19.2%)	223 (22.3%)
More than once a week	156 (31.2%)	168 (33.6%)	324 (32.4%)
Less than once a week	142 (28.4%)	168 (33.6%)	310 (31.0%)
Rarely or never	75 (15.0%)	68 (13.6%)	143 (14.3%)
Legumes			
Daily	22 (4.4%)	22 (4.4%)	44 (4.4%)
More than once a week	234 (39.4%)	271 (54.2%)	505 (50.5%)
Less than once a week	197 (39.4%)	185 (37.0%)	382 (38.2%)
Rarely or never	47 (9.4%)	22 (4.4%)	69 (6.9%)
Alcohol			
Daily	38 (7.6%)	83 (16.6%)	121 (12.1%)
More than once a week	95 (19.0%)	140 (28.0%)	235 (23.5%)
Less than once a week	136 (27.2%)	120 (24.0%)	256 (25.6%)
Rarely or never	231 (46.2%)	157 (31.4%)	388 (38.8%)

	Female<br>*n*=500 (%)	Male<br>*n*=500 (%)	Total<br>*N*=1,000 (%)
**Animal-based food items**			
Dairy and products			
Daily	337 (67.4%)	316 (63.2%)	653 (65.3%)
More than once a week	111 (22.2%)	129 (25.8%)	240 (24.0%)
Less than once a week	23 (4.6%)	25 (5.0%)	48 (4.8%)
Rarely or never	29 (5.8%)	30 (6.0%)	59 (5.9%)
Butter / margarine			
Daily	233 (46.6%)	235 (47.0%)	468 (46.8%)
More than once a week	125 (25.0%)	126 (25.2%)	251 (25.1%)
Less than once a week	83 (16.6%)	75 (15.0%)	158 (15.8%)
Rarely or never	59 (11.8%)	64 (12.8%)	123 (12.3%)
Pastries and sweets			
Daily	58 (11.6%)	52 (10.4%)	110 (11.0%)
More than once a week	169 (33.8%)	211 (42.2%)	380 (38.0%)
Less than once a week	188 (37.6%)	164 (32.8%)	352 (35.2%)
Rarely or never	85 (17.0%)	73 (14.6%)	158 (15.8%)
Red meat and products			
Daily	22 (4.4%)	19 (3.8%)	41 (4.1%)
More than once a week	195 (39.0%)	247 (49.4%)	442 (44.2%)
Less than once a week	197 (39.4%)	195 (39.0%)	392 (39.2%)
Rarely or never	86 (17.2%)	39 (7.8%)	125 (12.5%)
Poultry (not fried)			
Daily	18 (3.6%)	8 (1.6%)	26 1.6%)
More than once a week	230 (46.0%)	218 (43.6%)	448 (44.8%)
Less than once a week	188 (37.6%)	219 (43.8%)	407 (40.7%)
Rarely or never	64 (12.8%)	55 (11.0%)	119 (11.9%)
Fish (not fried)			
Daily	22 (4.4%)	8 (1.6%)	30 (3.0%)
More than once a week	184 (36.8%)	198 (39.6%)	382 (38.2%)
Less than once a week	222 (44.4%)	225 (45.0%)	447 (44.7%)
Rarely or never	72 (14.4%)	69 (13.8%)	141 (14.1%)
Fast and fried food			
Daily	3 (0.6%)	4 (0.8%)	7 (0.7%)
More than once a week	30 (6.0%)	43 (8.6%)	73 (7.3%)
Less than once a week	145 (29.0%)	177 (35.4%)	322 (32.2%)
Rarely or never	322 (32.2%)	276 (55.2%)	598 (59.8%)

Table S4: Association between dietary patterns and age groups.

	60 – 64 years<br>*n*=329 (%)	65 – 74 years<br>*n*=518 (%)	75+ years<br>*n*=153 (%)	Total<br>*N*=1,000 (%)
**Plant-based food items**				
Fruits and berries				
Daily	166 (50.5%)	285 (55.0%)	99 (64.7%)	550 (55.0%)
More than once a week	102 (31.0%)	172 (33.2%)	39 (25.5%)	313 (31.3%)
Less than once a week	48 (14.6%)	50 (9.7%)	13 (8.5%)	111 (11.1%)
Rarely or never	13 (4.0%)	11 (2.1%)	2 (1.3%)	26 (2.6%)
Vegetables				
Daily	138 (41.9%)	223 (43.1%)	75 (49.0%)	436 (43.6%)
More than once a week	153 (46.5%)	239 (46.1%)	65 (42.5%)	457 (45.7%)
Less than once a week	23 (7.0%)	52 (10.0%)	11 (7.2%)	86 (8.6%)
Rarely or never	15 (4.6%)	4 (0.8%)	2 (1.3%)	21 (2.1%)
Vegetable oils				
Daily	142 (43.2%)	227 (43.8%)	73 (47.7%)	442 (44.2%)
More than once a week	132 (40.1%)	198 (38.2%)	56 (36.6%)	386 (38.6%)
Less than once a week	36 (10.9%)	67 (12.9%)	19 (12.4%)	122 (12.2%)
Rarely or never	19 (5.8%)	26 (5.0%)	5 (3.3%)	50 (5.0%)
(Whole) grains and products				
Daily	90 (27.4%)	129 (24.9%)	37 (24.2%)	256 (25.6%)
More than once a week	127 (38.6%)	205 (39.6%)	57 (37.3%)	389 (38.9%)
Less than once a week	59 (17.9%)	97 (18.7%)	36 (23.5%)	192 (19.2%)
Rarely or never	53 (16.1%)	87 (16.8%)	23 (15.0%)	163 (16.3%)
Nuts and seeds				
Daily	70 (21.3%)	122 (23.6%)	31 (20.3%)	223 (22.3%)
More than once a week	98 (29.8%)	176 (34.0%)	50 (32.7%)	324 (32.4%)
Less than once a week	105 (31.9%)	152 (29.3%)	53 (34.6%)	310 (31.0%)
Rarely or never	56 (17.0%)	68 (13.1%)	19 (12.4%)	143 (14.3%)
Legumes				
Daily	17 (5.2%)	20 (3.9%)	7 (4.6%)	44 (4.4%)
More than once a week	166 (50.5%)	264 (51.0%)	75 (49.0%)	505 (50.5%)
Less than once a week	116 (35.3%)	204 (39.4%)	62 (40.5%)	382 (38.2%)
Rarely or never	30 (9.1%)	30 (5.8%)	9 (5.9%)	69 (6.9%)
Alcohol				
Daily	32 (9.7%)	67 (12.9%)	22 (14.4%)	121 (12.1%)
More than once a week	65 (19.8%)	125 (24.1%)	45 (29.4%)	235 (23.5%)
Less than once a week	88 (26.7%)	137 (26.4%)	31 (20.3%)	256 (25.6%)
Rarely or never	144 (43.8%)	189 (36.5%)	55 (35.9%)	388 (38.8%)

	60 – 64 years *n*=329 (%)	65 – 74 years *n*=518 (%)	75+ years *n*=153 (%)	Total *N*=1,000 (%)
**Animal-based food items**				
Dairy and products	221 (67.2%)	332 (64.1%)	100 (65.4%)	653 (65.3%)
Daily	73 (22.2%)	127 (24.5%)	40 (26.1%)	240 (24.0%)
More than once a week	14 (4.3%)	30 (5.8%)	4 (2.6%)	48 (4.8%)
Less than once a week	21 (6.4%)	29 (5.6%)	9 (5.9%)	59 (5.9%)
Rarely or never				
Butter / margarine				
Daily	141 (42.9%)	244 (47.1%)	83 (54.2%)	468 (46.8%)
More than once a week	84 (25.5%)	138 (26.6%)	29 (19.0%)	251 (25.1%)
Less than once a week	61 (18.5%)	74 (14.3%)	23 (15.0%)	158 (15.8%)
Rarely or never	43 (13.1%)	62 (12.0%)	18 (11.8%)	123 (12.3%)
Pastries and sweets				
Daily	36 (10.9%)	56 (10.8%)	18 (11.8%)	110 (11.0%)
More than once a week	117 (35.6%)	200 (38.6%)	63 (41.2%)	380 (38.0%)
Less than once a week	119 (36.2%)	184 (35.5%)	49 (32.0%)	352 (35.2%)
Rarely or never	57 (17.3%)	78 (15.1%)	23 (15.0%)	158 (15.8%)
Red meat and products				
Daily	16 (4.9%)	20 (3.9%)	5 (3.3%)	41 (4.1%)
More than once a week	138 (41.9%)	222 (42.9%)	82 (53.6%)	442 (44.2%)
Less than once a week	125 (38.0%)	214 (41.3%)	53 (34.6%)	392 (39.2%)
Rarely or never	50 (15.2%)	62 (12.0%)	13 (8.5%)	125 (12.5%)
Poultry (not fried)				
Daily	11 (3.3%)	12 (2.3%)	3 (2.0%)	26 1.6%)
More than once a week	153 (46.5%)	232 (44.8%)	63 (41.2%)	448 (44.8%)
Less than once a week	125 (38.0%)	210 (40.5%)	72 (47.1%)	407 (40.7%)
Rarely or never	40 (12.2%)	64 (12.4%)	15 (9.8%)	119 (11.9%)
Fish (not fried)				
Daily	10 (3.0%)	16 (3.1%)	4 (2.6%)	30 (3.0%)
More than once a week	124 (37.7%)	192 (37.1%)	66 (43.1%)	382 (38.2%)
Less than once a week	134 (40.7%)	245 (47.3%)	68 (44.4%)	447 (44.7%)
Rarely or never	61 (18.5%)	65 (12.5%)	15 (9.8%)	141 (14.1%)
Fast and fried food				
Daily	4 (1.2%)	2 (0.4%)	1 (0.7%)	7 (0.7%)
More than once a week	28 (8.5%)	36 (6.9%)	9 (5.9%)	73 (7.3%)
Less than once a week	111 (33.7%)	170 (32.8%)	41 (26.8%)	322 (32.2%)
Rarely or never	186 (56.5%)	310 (59.8%)	102 (66.7%)	598 (59.8%)

Table S5: Association between dietary patterns and countries.

	Germany *n*=250 (%)	Switzerland *n*=250 (%)	Spain n=250 (%)	France *n*=250(%)	Total *N*=1,000 (%)
**Plant-based food items**					
Fruits and berries					
Daily	127 (50.8%)	116 (46.4%)	176 (70.4%)	131 (52.4%)	550 (55.0%)
More than once a week	94 (37.6%)	88 (35.2%)	48 (19.2%)	83 (33.2%)	313 (31.3%)
Less than once a week	24 (9.6%)	39 (15.6%)	20 (8.0%)	28 (11.2%)	111 (11.1%)
Rarely or never	5 (2.0%)	7 (2.8%)	6 (2.4%)	8 (3.2%)	26 (2.6%)
Vegetables					
Daily	81 (32.4%)	122 (48.8%)	113 (45.2%)	120 (48.0%)	436 (43.6%)
More than once a week	129 (51.6%)	108 (43.2%)	115 (46.0%)	105 (42.0%)	457 (45.7%)
Less than once a week	34 (13.6%)	15 (6.0%)	20 (8.0%)	17 (6.8%)	86 (8.6%)
Rarely or never	6 (2.4%)	5 (2.0%)	2 (0.8%)	8 (3.2%)	21 (2.1%)
Vegetable oils					
Daily	71 (28.4%)	107 (42.8%)	171 (68.4%)	93 (37.2%)	442 (44.2%)
More than once a week	121 (48.4%)	97 (38.8%)	59 (23.6%)	109 (43.6%)	386 (38.6%)
Less than once a week	43 (17.2%)	31 (12.4%)	9 (3.6%)	39 (15.6%)	122 (12.2%)
Rarely or never	15 (6.0%)	15 (6.0%)	11 (4.4%)	9 (3.6%)	50 (5.0%)
(Whole) grains and products					
Daily	89 (35.6%)	71 (28.4%)	52 (20.8%)	44 (17.6%)	256 (25.6%)
More than once a week	133 (53.2%)	113 (45.2%)	70 (28.0%)	73 (29.2%)	389 (38.9%)
Less than once a week	21 (8.4%)	48 (19.2%)	67 (26.8%)	56 (22.4%)	192 (19.2%)
Rarely or never	7 (2.8%)	18 (7.2%)	61 (24.4%)	77 (30.8%)	163 (16.3%)
Nuts and seeds					
Daily	38 (15.2%)	67 (26.8%)	73 (29.2%)	45 (18.0%)	223 (22.3%)
More than once a week	85 (34.0%)	76 (30.4%)	95 (38.0%)	68 (27.2%)	324 (32.4%)
Less than once a week	88 (35.2%)	75 (30.0%)	56 (22.4%)	91 (36.4%)	310 (31.0%)
Rarely or never	39 (15.6%)	32 (12.8%)	26 (10.4%)	46 (18.4%)	143 (14.3%)
Legumes					
Daily	8 (3.2%)	5 (2.0%)	10 (4.0%)	21 (8.4%)	44 (4.4%)
More than once a week	86 (34.4%)	105 (42.0%)	187 (74.8%)	127 (50.8%)	505 (50.5%)
Less than once a week	127 (50.8%)	112 (44.8%)	51 (20.4%)	92 (36.8%)	382 (38.2%)
Rarely or never	29 (11.6%)	28 (11.2%)	2 (0.8%)	10 (4.0%)	69 (6.9%)
Alcohol					
Daily	20 (8.0%)	41 (16.4%)	23 (9.2%)	37 (14.8%)	121 (12.1%)
More than once a week	58 (23.2%)	71 (28.4%)	56 (22.4%)	50 (20.0%)	235 (23.5%)
Less than once a week	54 (21.6%)	60 (24.0%)	67 (26.8%)	75 (30.0%)	256 (25.6%)
Rarely or never	118 (47.2%)	78 (31.2%)	104 (41.6%)	88 (35.2%)	388 (38.8%)

	Germany *n*=250 (%)	Switzerland *n*=250 (%)	Spain n=250 (%)	France *n*=250(%)	Total *N*=1,000 (%)
**Animal-based food items**					
Dairy and products	126 (50.4%)	166 (66.4%)	199 (79.6%)	162 (64.8%)	653 (65.3%)
Daily	93 (37.2%)	51 (20.4%)	31 (12.4%)	65 (26.0%)	240 (24.0%)
More than once a week	14 (5.6%)	15 (6.0%)	4 (1.6%)	15 (6.0%)	48 (4.8%)
Less than once a week	17 (6.8%)	18 (7.2%)	16 (6.4%)	8 (3.2%)	59 (5.9%)
Rarely or never					
Butter / margarine					
Daily	165 (66.0%)	128 (51.2%)	43 (17.2%)	132 (52.8%)	468 (46.8%)
More than once a week	56 (22.4%)	70 (28.0%)	63 (25.2%)	62 (24.8%)	251 (25.1%)
Less than once a week	12 (4.8%)	30 (12.0%)	75 (30.0%)	41 (16.4%)	158 (15.8%)
Rarely or never	17 (6.8%)	22 (8.8%)	69 (27.6%)	15 (6.0%)	123 (12.3%)
Pastries and sweets					
Daily	42 (16.8%)	35 (14.0%)	16 (6.4%)	17 (6.8%)	110 (11.0%)
More than once a week	107 (42.8%)	129 (51.6%)	64 (25.6%)	80 (32.0%)	380 (38.0%)
Less than once a week	76 (30.4%)	62 (24.8%)	103 (41.2%)	111 (44.4%)	352 (35.2%)
Rarely or never	25 (10.0%)	24 (9.6%)	67 (26.8%)	42 (16.8%)	158 (15.8%)
Red meat and products					
Daily	15 (6.0%)	9 (3.6%)	8 (3.2%)	9 (3.6%)	41 (4.1%)
More than once a week	101 (40.4%)	117 (46.8%)	105 (42.0%)	119 (47.6%)	442 (44.2%)
Less than once a week	91 (36.4%)	96 (38.4%)	114 (45.6%)	91 (36.4%)	392 (39.2%)
Rarely or never	43 (34.4%)	28 (22.4%)	23 (18.4%)	31 (12.4%)	125 (12.5%)
Poultry (not fried)					
Daily	1 (0.4%)	6 (2.4%)	8 (3.2%)	11 (4.4%)	26 1.6%)
More than once a week	72 (28.8%)	81 (32.4%)	162 (64.8%)	133 (53.2%)	448 (44.8%)
Less than once a week	133 (53.2%)	126 (50.4%)	59 (23.6%)	89 (35.6%)	407 (40.7%)
Rarely or never	44 (17.6%)	37 (14.8%)	21 (8.4%)	17 (6.8%)	119 (11.9%)
Fish (not fried)					
Daily	0 (0.0%)	6 (2.4%)	16 (6.4%)	8 (3.2%)	30 (3.0%)
More than once a week	52 (20.8%)	66 (26.4%)	145 (58.0%)	119 (47.6%)	382 (38.2%)
Less than once a week	140 (56.0%)	136 (54.4%)	71 (28.4%)	100 (40.0%)	447 (44.7%)
Rarely or never	58 (23.2%)	42 (16.8%)	18 (7.2%)	23 (9.2%)	141 (14.1%)
Fast and fried food					
Daily	0 (0.0%)	3 (1.2%)	1 (0.4%)	3 (1.2%)	7 (0.7%)
More than once a week	15 (6.0%)	5 (2.0%)	44 (17.6%)	9 (3.6%)	73 (7.3%)
Less than once a week	84 (33.6%)	64 (25.6%)	94 (37.6%)	80 (32.0%)	322 (32.2%)
Rarely or never	151 (60.4%)	178 (71.2%)	111 (44.4%)	158 (63.2%)	598 (59.8%)

Table S6: Mean of consumption across the countries.

	Germany	Switzerland	Spain	France
**Food Items**				
Fruits and berries	1.63	1.75	1.42	1.65
Vegetables	1.86	1.61	1.64	1.65
Vegetable oils	2.01	1.82	1.44	1.86
(Whole) grains and products	1.78	2.05	2.55	2.66
Nuts and seeds	2.51	2.29	2.14	2.55
Legumes	2.71	2.65	2.18	2.36
Alcohol	3.08	2.70	3.01	2.86
Dairy and products	1.69	1.54	1.35	1.48
Butter / margarine	1.52	1.78	2.68	1.76
Pastries and sweets	2.34	2.30	2.88	2.71
Red meat and products	2.65	2.57	2.61	2.58
Poultry (not fried)	2.88	2.78	2.37	2.45
Fish (not fried)	3.02	2.86	2.36	2.55
Fast food or fried food	3.54	3.67	3.26	3.57

Figure S1: Questionnaire (General English version).

A survey on nutrition and nutrition communication in older age

Dear Sir or Madam,

thank you for your interest in our survey. You are invited to participate in a **research project on communication strategies on nutritional behaviour in adults aged 60 years and over** by completing the following questionnaire.

This project is part of the European project 'ITN SmartAge', which is investigating the connection between the gut and the brain and what influence diet and other factors can have.

The aim of this survey is to find out more about your needs, knowledge, interests and attitudes towards nutrition and nutrition communication.

The questionnaire will take about 10-15 minutes to complete. Participation in the survey and answering the questions is voluntary and anonymous. Your data will be kept strictly confidential and will only be used for scientific purposes. Please take your time and select the answers that best apply to you.

Thank you for taking the time to participate in this survey! To begin the survey, please scroll down and click 'Continue'.

Kind regards,
Julia Juber

If you have any further questions, please contact us via the following contact information:

Julia Juber
[Contact information]

Prof. Dr. Christine Brombach
[Contact information]

Prof. Dr. Christel Rademacher
[Contact information]

Consent for data collection

By selecting "Yes I agree", you approve to the following:

- I am at least 60 years or older.
- I have been informed about the study and I have read the written information.
- I have had the opportunity to think about my participation in this study and it is entirely voluntary.
- I have the right at any time to withdraw the permission I give and quit my participation in this study, without giving reasons.

◯ Yes, I agree.

◯ No, I do not agree.

How old are you? (Please type in your age in numbers.)

______________________ **Years**

What is your gender? (Please choose your appropriate answer.)

◯ Female
◯ Male
◯ Other or prefer not to indicate

What is the highest level of education you achieved? (Please choose your appropriate answer.)

◯ The answer options varied according to the education system from each country

To begin this survey, we would like to find out more about your usage of different information channels and your nutrition needs.

Which information sources do you use regularly? (Please choose your appropriate answers.)

☐ Television
☐ Radio
☐ Internet or social media (e.g., Google, YouTube, Instagram, Twitter, Facebook, …)
☐ Newspaper, Books, magazines, brochures, ...
☐ Family, friends, neighbours, ...
☐ Doctor (all medical fields) or Dietitian/Nutritionist
☐ Pharmacies
☐ Official Institutions (e.g., care centres, national nutrition associations, consumer associations, ...)
☐ None of these

Do you inform yourself about nutrition? (Please choose your appropriate answer.)

◯ Yes, I inform myself actively.
◯ I get information by coincidence or passively
◯ No, I don't inform myself.

How difficult is it for you to obtain information about nutrition? (Please choose your appropriate answer.)

◯	◯	◯	◯	◯
very difficult	rather difficult	medium	rather easy	very easy

→ Filter: Only the information sources selected in Question 3 will be displayed.

How much do you rely on the nutrition and health information from the following sources? (Please choose your appropriate answer for each source.)

	Very low	Low	Medium	High	Very high
Television	◯	◯	◯	◯	◯
Radio	◯	◯	◯	◯	◯
Internet or social media (e.g., Google search, YouTube, Instagram, Twitter, Facebook, ...)	◯	◯	◯	◯	◯
Newspaper, Books, magazines, brochures, ...	◯	◯	◯	◯	◯
Family, friends, neighbours, ...	◯	◯	◯	◯	◯
Doctor (all medical fields) and/or Dietitian/Nutritionist	◯	◯	◯	◯	◯
Pharmacies	◯	◯	◯	◯	◯
Official Institutions (e.g., care centres, national nutrition associations, consumer associations, ...)	◯	◯	◯	◯	◯

How much do you think that your diet impacts your health status? (Please choose your appropriate answer.)

0 **1** **2** **3** **4** **5**

No impact — Strong impact

Have you ever been advised by a health care professional to have a healthier diet? (Please choose your appropriate answer.)

◯ Yes ◯ No

Do you want to have a healthier diet? (Please choose your appropriate answer.)

- ◯ Yes, I want to change my diet.
- ◯ No, I am fine with my diet.
- ◯ I have already successfully changed my diet.

→ Filter: The following question will only be displayed, if «Yes, I want to change my diet.» have been chosen.

Do you need support to have a healthier diet? (Please choose your appropriate answer.)

- ◯ Yes, I need support.
- ◯ No, I do not need support.

→ Filter: The following question will only be displayed, if « Yes, I need support.» have been chosen.

What kind of support would you like to receive? (Please choose your appropriate answer/s.)

- ☐ More social support from partner, family, friends, ...
- ☐ More advice from official health centres
- ☐ More advice from my doctors, dietitian/nutritionist, pharmacist, ...
- ☐ More freely accessible information
- ☐ Staying motivated
- ☐ (open answer) ____________________

The upcoming questions are related to your interests and your daily lifestyle.

In which topics are you most interested in to obtain information? (Please choose your appropriate answers.)

- ☐ Specific requirements at older age
- ☐ Preparing and planning healthy meals/snacks
- ☐ Dieting/weight loss
- ☐ Weight gain
- ☐ Sustainability/Regionality/Seasonality
- ☐ Replace/Avoid specific food items
- ☐ Eat healthy on a lower budget
- ☐ Healthy and balanced recipes
- ☐ Diet to improve certain diseases (e.g., diabetes, dementia, cardiovascular diseases)
- ☐ Increase water intake
- ☐ None of these

What are your TOP 3 motives to pursue a healthier diet? (Please choose your appropriate 3 answers.)

- ☐ Being healthier
- ☐ Being more active & efficient
- ☐ Well-being
- ☐ Being more environmentally friendly
- ☐ Prevent further diseases and disability
- ☐ Living a longer life
- ☐ None of these
- ☐ I do not want to eat healthier

What kind of activities do you do regularly? (Please choose your appropriate answers.)

- ☐ Reading (e.g., newspaper, magazines, books, ...)
- ☐ Games (e.g., card games, board games, puzzles, memory games, chess, ...)
- ☐ Crossword or sudoku
- ☐ Painting or drawing
- ☐ Meeting with friends, family, neighbours, ...
- ☐ Gardening
- ☐ Leisure sports or club sports (e.g., fitness center, badminton, soccer, boule games, skittles, ...)
- ☐ Walking or hiking
- ☐ Riding a bike
- ☐ None of these

How often do you consume the following plant-based food items? (Please choose your appropriate answer for each item.)

	Daily	More than once a week	Less than once a week	Rarely or never
Vegetables (also potatoes, peas, tomato sauce or string beans)	○	○	○	○
Legumes (e.g., beans, lentils, soybeans, ...)	○	○	○	○
Fruits and berries (e.g., bananas, grapes, kiwis, oranges, strawberries, rasberries, ...)	○	○	○	○
Nuts and seeds	○	○	○	○
Vegetable oils	○	○	○	○
Whole grains (e.g., whole wheat bread, brown rice, oatmeal, ...)	○	○	○	○

Alcohol	◯	◯	◯	◯

How often do you consume the following animal-based food items? (Please choose your appropriate answer for each item.)

	Daily	More than once a week	Less than once a week	Rarely or never
Dairy and dairy products	◯	◯	◯	◯
Butter or margarine	◯	◯	◯	◯
Fish (not fried)	◯	◯	◯	◯
Poultry (not fried)	◯	◯	◯	◯
Red meat and products	◯	◯	◯	◯
Pastries and sweets	◯	◯	◯	◯
Fast food or fried food	◯	◯	◯	◯

How much do the following statements apply to you? (Please choose your appropriate answer for each item.)

	Doesn't apply at all	Applies a bit	Applies somewhat	Applies mostly	Applies completely
I can rely on my own abilities in difficult situations.	◯	◯	◯	◯	◯
I am able to solve most problems on my own.	◯	◯	◯	◯	◯
I can usually solve even challenging and complex tasks well.	◯	◯	◯	◯	◯

Do you know the national consumption recommendations? (Please choose your appropriate answer.)

[Photo adapted to national consumption recommendations]

◯ Yes

◯ No

Are the following foods a good source of fibre per portion? (Please choose your appropriate answer.)

	Yes	No	I do not know
Wheat bread	◯	◯	◯
Potatoes	◯	◯	◯
Wholegrain bread	◯	◯	◯
Legumes	◯	◯	◯

Red meat	◯	◯	◯

Lastly, we want to know more about yourself and your living conditions.

How would you describe your current state of health in general? (Please choose your appropriate answer.)

◯	◯	◯	◯	◯	◯
Very bad	Bad	Medium	Good	Very good	Prefer not to indicate

In which country do you live? (Please choose your appropriate answer.)

- ◯ Germany
- ◯ Switzerland
- ◯ Spain
- ◯ France

How would you describe the general area where you live? (Please choose your appropriate answer.)

- ◯ Urban or suburban
- ◯ Rural

How many people live in your household including yourself? (Please choose your appropriate answer.)

◯	◯	◯	◯	◯	◯
1	2	3	4	5	6 or more

Are you responsible for cooking in your household? (Please choose your appropriate answer.)

- ◯ Yes
- ◯ No
- ◯ Shared responsibility
- ◯ I receive my meals from an external provider

Are you responsible for grocery shopping in your household? (Please choose your appropriate answer.)

- ◯ Yes
- ◯ No
- ◯ Shared responsibility
- ◯ I receive external help, e.g., from another person

Where do you go for grocery shopping regularly? (Please choose your appropriate answers.)

- [ ] Specific grocery store (e.g., bakery, butcher, greengrocer, fishmonger, ...)
- [ ] Retail trade (e.g., EDEKA, Aldi, Lidl, NORMA, Penny, ...)
- [ ] Heathy/biological food store
- [ ] Weekly market, direct marketer, producer
- [ ] None of these

Thank you for completing the questionnaire!

We would like to thank you very much for helping us. Your answers were transmitted, you may close the browser window or tab now.

If you have any further questions, please contact us via the following contact information:

Julia Juber

[Contact information]

Prof. Dr. Christine Brombach

[Contact information]

Prof. Dr. Christel Rademacher

[Contact information]

This research received funding by the Marie Skłodowska-Curie Innovative Training Network SmartAge funded by the Horizon 2020 Framework Programme of the European Union under grant agreement number 859890.
